# Can cancer researchers accurately judge whether preclinical reports will reproduce?

**DOI:** 10.1371/journal.pbio.2002212

**Published:** 2017-06-29

**Authors:** Daniel Benjamin, David R. Mandel, Jonathan Kimmelman

**Affiliations:** 1 Biomedical Ethics Unit/STREAM, McGill University, Montreal, Canada; 2 York University, Department of Psychology, Toronto, Canada; University of Sydney, Australia

## Abstract

There is vigorous debate about the reproducibility of research findings in cancer biology. Whether scientists can accurately assess which experiments will reproduce original findings is important to determining the pace at which science self-corrects. We collected forecasts from basic and preclinical cancer researchers on the first 6 replication studies conducted by the Reproducibility Project: Cancer Biology (RP:CB) to assess the accuracy of expert judgments on specific replication outcomes. On average, researchers forecasted a 75% probability of replicating the statistical significance and a 50% probability of replicating the effect size, yet none of these studies successfully replicated on either criterion (for the 5 studies with results reported). Accuracy was related to expertise: experts with higher h-indices were more accurate, whereas experts with more topic-specific expertise were less accurate. Our findings suggest that experts, especially those with specialized knowledge, were overconfident about the RP:CB replicating individual experiments within published reports; researcher optimism likely reflects a combination of overestimating the validity of original studies and underestimating the difficulties of repeating their methodologies.

## Author summary

Science is supposed to be self-correcting. However, the efficiency with which science self-corrects depends in part on how well scientists can anticipate whether particular findings will hold up over time. We examined whether expert researchers could accurately forecast whether mouse experiments in 6 prominent preclinical cancer studies conducted by the Reproducibility Project: Cancer Biology would reproduce original effects. Experts generally overestimated the likelihood that replication studies would reproduce the effects observed in original studies. Experts with greater publication impact (as measured by h-index) provided more accurate forecasts, but experts did not consistently perform better than trainees, and topic-specific expertise did not improve forecast skill. Our findings suggest that experts tend to overestimate the reproducibility of original studies and/or they underappreciate the difficulty of independently repeating laboratory experiments from original protocols.

## Introduction

Approximately 90% of new drugs entered into clinical development on promising preclinical findings fail to yield sufficient efficacy and safety to receive a Food and Drug Administration (FDA) license [1]. Such high rates of discordance between preclinical reports and clinical effects have prompted mounting scrutiny of the design, reporting, and reproducibility of preclinical research.

Being unable to reproduce preclinical findings can impede medical progress and misallocate intellectual capital. For example, laboratories attempting to reproduce original findings might squander limited research resources because they are not attuned to subtle techniques needed to reproduce an original finding. Of even greater concern is the prospect of launching clinical investigations on the backs of invalid preclinical reports. Scientists’ judgments about an experiment’s reproducibility influence how they go about designing similar studies, as well as which findings are utilized for the design of subsequent studies. Accurate judgment about reproducibility therefore affects how efficiently science self-corrects.

There is widespread debate about the scope of the reproducibility problem in many scientific fields, including biomedical research. Most scientists (52%) think there is a “significant” reproducibility crisis, whereas the remainder think there is, at worst, a “slight” crisis [2]. Some direct efforts at reproducing preclinical and basic science findings have suggested that many original findings may be spurious. For example, researchers at Amgen were unable to reproduce 89% of cancer preclinical studies provided to them by academic scientists; similar challenges were reported by Bayer scientists [3]. However, community-wide beliefs are somewhat measured, since 31% of researchers believe a result still might be true even in light of a failed replication attempt [2]. Indeed, many commentators have questioned the efforts and expertise of those conducting replication studies [4,5]. Uncertainty about the extent, causes, and impact of the “reproducibility crisis” has led to calls for more systematic assessments of the problem [6]. The Reproducibility Project: Cancer Biology (RP:CB) is currently conducting 29 direct replication studies to evaluate the replication rate in cancer biology [7].

By collecting subjective probability forecasts on outcomes that can be unambiguously dichotomized, one can score the accuracy of individuals’ strength of belief using well-established methods from the decision sciences [8]. For example, in a study examining economists’ predictive judgments about replication studies, forecasts significantly correlated with replication results and beat a matched prediction market about the same replication studies [9]. In contrast, psychologists’ forecasts did not significantly correlate with replication results, and their forecasts were beaten by a similar, matched prediction market [10].

Here, we report forecasts and their accuracy for the first 6 mouse-model replication studies conducted by the RP:CB. We set out primarily to determine the extent to which expert cancer researchers can accurately predict the probability of replicating significance levels and effect sizes from specific original studies in the RP:CB. We close by discussing our findings in light of the many complexities of conducting and interpreting replication studies, including the particular experiments used in our surveys.

## Results

### Sample demographics

We recruited a sample of 196 participants, including 138 experts and 58 novices, to perform forecasts for our study (Table 1 summarizes the sample demographics). Experts were predominantly male, whereas there were slightly more female than male novices. Our expert sample showed a somewhat lower mean h-index compared with nonresponders (25.6 versus 32.4)—based on an equally sized random sample of nonresponders.

**Table 1:** Sample demographics by rank of participants.

Variable	Rank	*N*	Mean	SD	Skew	Median	Min	Max
Age	Expert	134	47.0	11.4	0.4	45.5	20	81
% Male	Expert	133	80.5%					
h-index	Expert	130	25.6	20.2	1.7	20	0	113
% Editors	Expert	115	31.3%					
Age	Novice	56	29.3	5.5	0.8	29	19	47
% Male	Novice	58	44.8%					
Classes/Labs	Novice	45	19.2	18.4	2.4	16	0	100

“% Editors” refers to the percentage of individuals in the expert subsample who are or have been journal editors. “Classes/Labs” refers to the number of semesters spent in a cancer class or lab that studies cancer.

The average expert had published 89.5 (SD = 109.0) papers and been cited 4,546.1 (SD = 7,932.4) times. A third of the sample (27% of experts and 44% of novices) had not heard of the RP:CB, while 9% (10% of experts and 7% of novices) were actively awaiting results. The expert sample believed they had reasonable expertise pertaining to the studies in our survey. The mean expertise rating was 4.2 (SD = 1.9) on a 7-point scale (with 1 representing minimal expertise and 7 representing maximal expertise). A plurality of expert participants (45%) rated their expertise above the scale’s midpoint, and 33% below the midpoint. The mean expertise rating for the novices was 2.9 (SD = 1.5), with only 15% of novice expertise ratings above the midpoint and 65% below. Both the experts and novices rated high confidence in their forecasts, 77% versus 76% respectively. For both groups, 84% of confidence ratings were above 50%, and only 3% were below.

Table 2 shows the publications used in this forecasting study and summaries of forecasts for each [11–16]. Disease indications in xenograft studies included prostate, lung, renal cell, and breast cancer, as well as myeloma, leukemia, and melanoma, and the experiments used a variety of outcome measures, including disease-free survival, tumor weight and volume, organic phase absorbance, and bioluminescence. Studies were preselected by the RP:CB using a standardized search method [7].

**Table 2:** Study references and forecast sample sizes.

Study #	First Author	Title	Journal	Year	Significance		Effect Size	
					Expert	Novice	Expert	Novice
15	Sugahara	Coadministration of a tumor-penetrating peptide enhances the efficacy of cancer drugs	*Science*	2010	59	50	59	50
21	Sirota	Discovery and preclinical validation of drug indications using compendia of public gene expression data	*Science*	2011	59	53	59	53
44	Berger	Melanoma genome sequencing reveals frequent PREX2 mutations	*Nature*	2012	60	54	59	54
19	Delmore	BET bromodomain inhibition as a therapeutic strategy to target c-Myc	*Cell*	2011	72	N/A	71	N/A
29	Dawson	Inhibition of BET recruitment to chromatin as an effective treatment for MLL-fusion leukaemia	*Nature*	2011	70	N/A	69	N/A
39	Willingham	The CD47-signal regulatory protein alpha (SIRPa) interaction is a therapeutic target for human solid tumors	*PNAS*	2012	69	N/A	67	N/A

N/A, not applicable; *PNAS*, *Proceedings of the National Academy of Sciences*.

### Forecast properties

**Fig 1:**
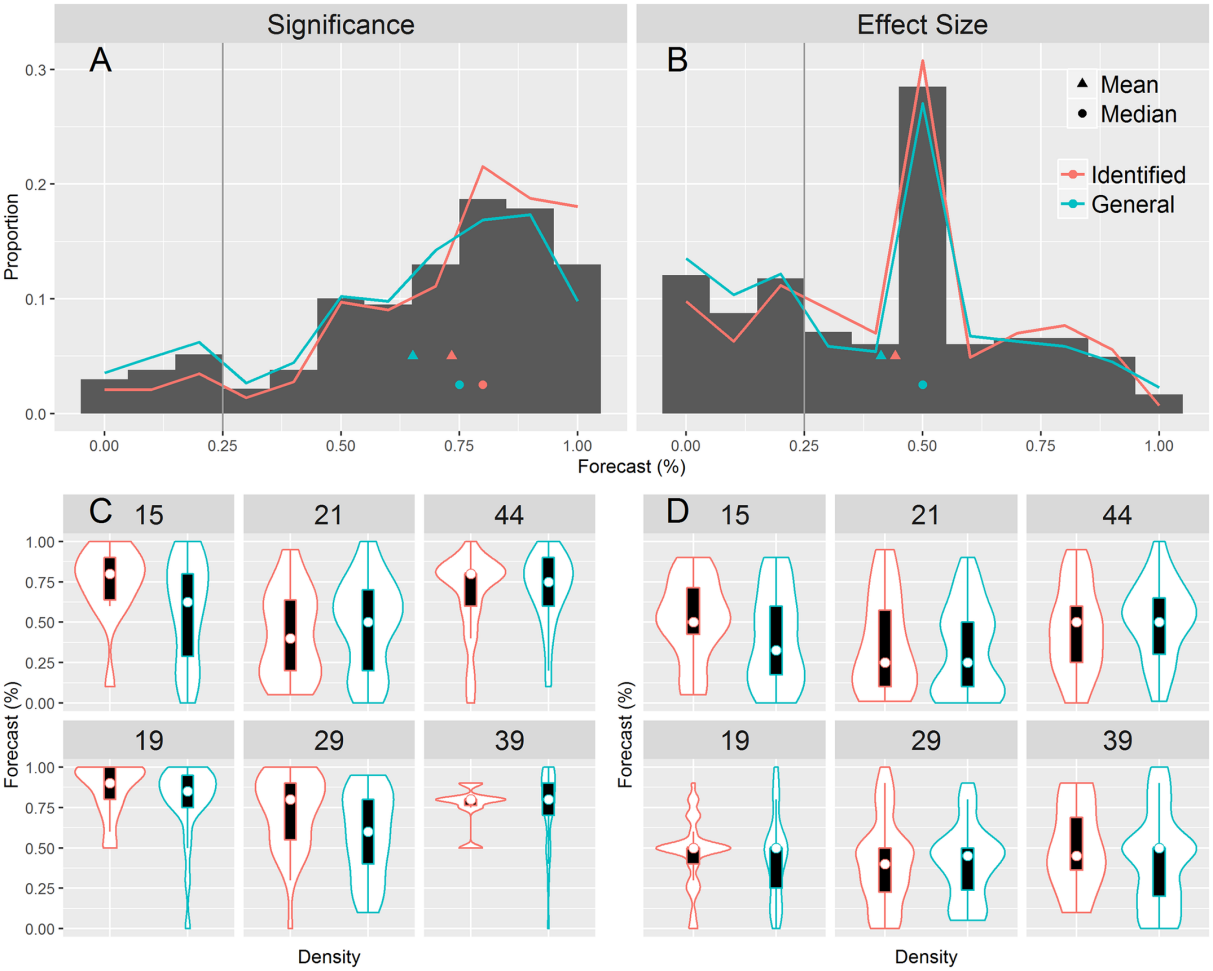
Expert forecasts by identified expertise and by study. The gray histogram represents the forecasts of significance (panel A) and effect size (panel B) with bins of 5%. A 100% forecast and a 0% forecast would indicate certainty that an event will occur and will not occur, respectively; 50% would indicate maximum uncertainty. The red lines represent forecasts for the studies in which each forecaster was identified during sampling via either citations or a “similar articles” search (“identified expertise”). The blue lines represent the forecasts made by experts on studies that did not lead to their identification in sampling (“general expertise”). The lower panels are violin (probability density) plots for each outcome type (panel C for significance and panel D for effect size) and each study by both identified (in red) and general (in blue) expertise. Box plots are contained within each violin plot.

Experts used the whole spectrum of belief (see Fig 1). They tended to believe replication studies would demonstrate statistical significance, since 73% of forecasts were above 50%, and 49% were above 75%. Only 18% of forecasts were below 50%. In contrast, experts gave lower forecasts (centered near 50%) for effect size, with forecast patterns suggesting community uncertainty (median forecast of 50% for 4 studies and the other 2 suggesting doubt (median forecasts of 25% and 40%).

Our question about effect sizes could be interpreted differently, resulting in high variability in forecasts. To corroborate interpretation of effect size forecasts, we asked forecasters the likelihood of a perfect replication study producing effect sizes as large or larger than the original, if an original finding were reproducible. We then compared these figures with an individual’s forecasts (see S3 Fig in the Supporting Information). A total of 17% of experts offered forecasts equal to their idealized replication, while 56% provided forecasts that were lower than their ideal, suggesting a degree of doubt about these studies and/or the RP:CB methods.

### Forecast accuracy

None of the 5 reproducibility studies in our survey successfully replicated the original studies using either significance or effect-size criteria [17–21]. Thus, accurate forecasting would require a strongly pessimistic response pattern. Table 3 displays experts’ forecast accuracy by outcome and expertise types. Experts were extremely overconfident about the replication rate, especially for significance. The specificity of all expert forecasts was 0.39, meaning 39% of forecasts (excluding fence-sitting 50% forecasts) were in the correct direction. The specificity of significance forecasts was 0.20, showing that the experts were substantially overoptimistic about significance replicability. The specificity of effect size forecasts was better, 0.64. Therefore, experts were more accurate (and pessimistic) about the replicability of effect sizes than significance levels.

**Table 3:** Forecast accuracy by outcome type, identified expertise, and rank.

Outcome	Expertise	Survey	*N*	Mean Brier	95% CI		Median Brier	Specificity
					Lower	Upper		
All	All	All	773	0.40	0.38	0.45	0.42	0.39
Significance			389	0.55	0.52	0.61	0.58	0.20
Effect Size			384	0.26	0.23	0.30	0.25	0.64
All	Identified	All	287	0.44	0.39	0.48	0.45	0.35
	General		447	0.38	0.35	0.42	0.41	0.42
Significance	Identified		144	0.61	0.54	0.66	0.64	0.13
	General		225	0.52	0.49	0.58	0.56	0.24
Effect Size	Identified		143	0.27	0.23	0.32	0.25	0.63
	General		222	0.25	0.21	0.29	0.24	0.65
All	Experts	Survey 1	355	0.34	0.30	0.39	0.32	0.43
Significance			178	0.43	0.38	0.49	0.42	0.30
Effect Size			177	0.25	0.21	0.30	0.25	0.59
All	Novices		314	0.38	0.35	0.46	0.35	0.45
Significance			157	0.51	0.48	0.60	0.50	0.24
Effect Size			157	0.25	0.21	0.33	0.17	0.68

95% CI refers to a bias-corrected bootstrap 95% confidence interval with 5,000 resamples.

**Fig 2:**
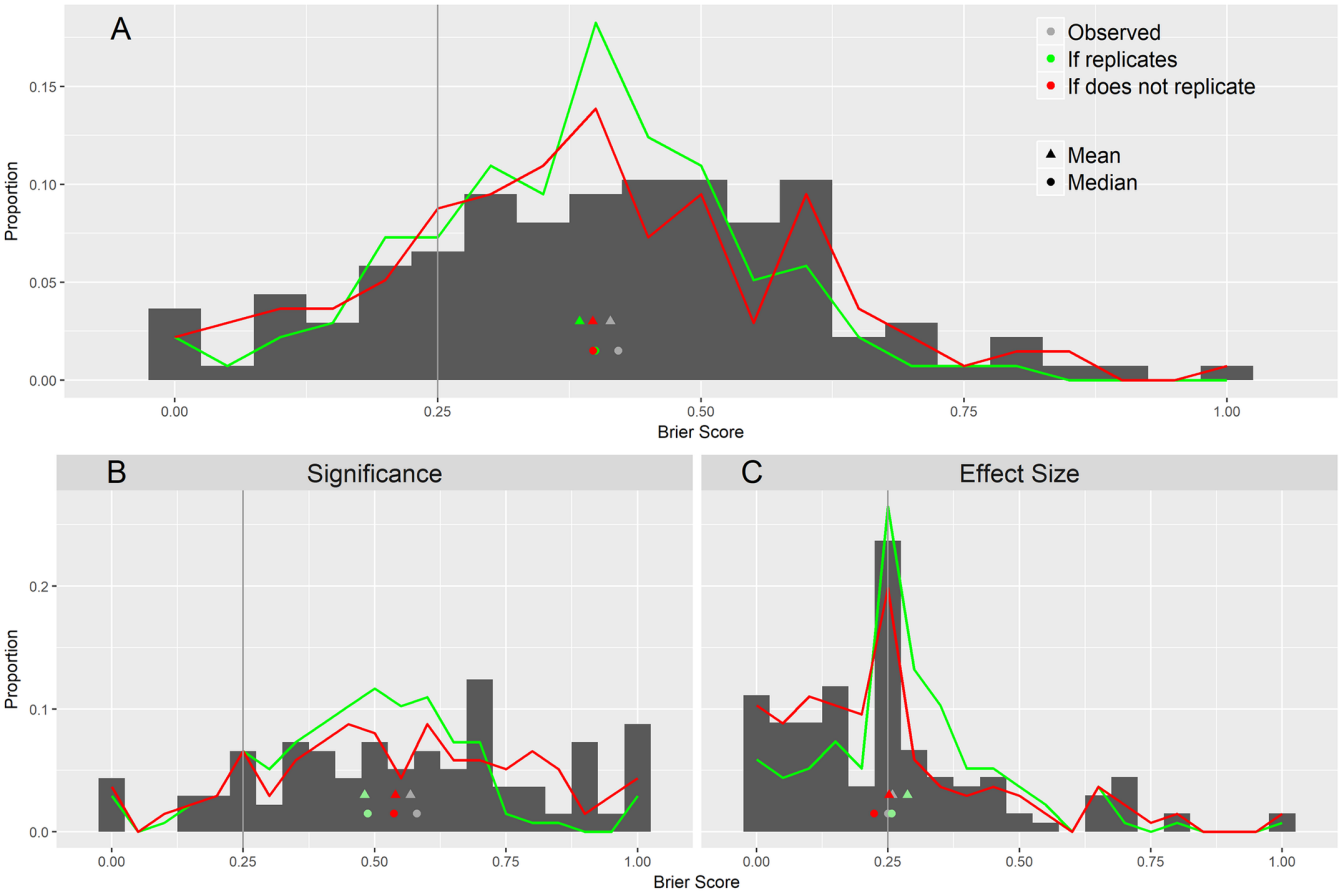
Brier scores by outcome type with sensitivity analysis. The gray histograms display Brier scores for the 5 studies with known results. All experts are displayed in panel A, and they are divided by outcome types in the bottom panels (B for significance and C for effect size). The green lines represent the probability density of Brier scores if the sixth study replicates each outcome, and the red line represents if it fails to replicate for each.

Forecasters’ mean Brier score was 0.40, 95% CI (0.38–0.45), indicating forecasting skill was significantly worse in our expert sample than guessing 50% each time (the latter would have generated a Brier score of 0.25) (see Fig 2). A fifth of experts (21%) had mean Brier scores less than 0.25. Among experts, Brier scores were worse for significance than for effect-size forecasts (median = 0.58 and 0.25, respectively), with 11% of experts scoring better than 0.25 (4% for significance forecasts and 30% for effect-size forecasts).

### Factors associated with greater forecasting skill

A possible explanation for low Brier scores is that forecasters might have lacked expertise in the specific studies in our sample. We probed the relationship between expertise and forecasting skill in several ways.

First, we tested whether forecasters performed better on the studies for which we had identified them as experts. Forecasts with identified expertise were more likely to use high extreme values (63% of forecasts were ≥ 75% compared to 51% with general expertise, a difference of 12%, 95% CI [0.02 to 0.22]) and less likely to give forecasts near the low extreme (8% of forecasts were ≤ 25% compared to 15% with general expertise, a difference of 7%, 95% CI [−0.13 to −0.002]), suggesting greater optimism about reproducibility in one’s own research area. The mean Brier score for significance forecasts was marginally greater for identified expertise (0.61, 95% CI [0.54 to 0.66]) than for general expertise (0.52, 95% CI [0.49 to 0.58] for a mean difference of 0.09, 95% CI [−0.01 to 0.15]). Effect-size forecasts centered around 50% (in terms of median and mode) for both identified and general expertise. Forecasts from general expertise were more likely to use extreme low values: 36% of forecasts in the lowest quartile compared to 27% for identified expertise (a difference of 9%, 95% CI [−0.01 to 0.18]). Experts performed better when they were not identified as having expertise, possibly indicating that experts are overconfident when they are more knowledgeable and/or more doubtful about others’ research.

A second way we probed the relationship between expertise and forecasting skill was by comparing experts to a sample of trainees in competitive molecular biology programs. We reasoned that if experts outperformed novices, then forecasting success is associated with expertise; if novices outperformed experts, then there is evidence that expertise is associated with overconfidence. We observed the same general patterns of (a) optimism for significance forecasts and (b) uncertainty for effect-size forecasts (centered around a mode of 50%) with a pessimistic leaning (a local mode at 10%) (see S2 Fig in the Supporting Information for a comparison of expert and novice forecasts). Overall performance did not favor either group: experts slightly outperformed novices in accuracy (median Brier = 0.32 versus 0.35), but novices slightly outperformed experts in specificity (0.45 versus 0.43). Experts’ significance forecasts were significantly more accurate (mean Brier score 0.43, 95% CI [0.38 to 0.49]) as compared with novices (mean = 0.51, 95% CI [0.48 to 0.60] for a mean difference of 0.08, 95% CI [−0.18 to −0.02]) and showed better specificity (0.30 versus 0.24). Experts were more pessimistic (i.e., more likely to offer extremely low forecasts and less likely to use extremely high forecasts). We observed the opposite pattern for effect-size forecasts. Median Brier scores (0.17 versus 0.25) and specificity (0.68 versus 0.59) for novices beat experts.

**Fig 3:**
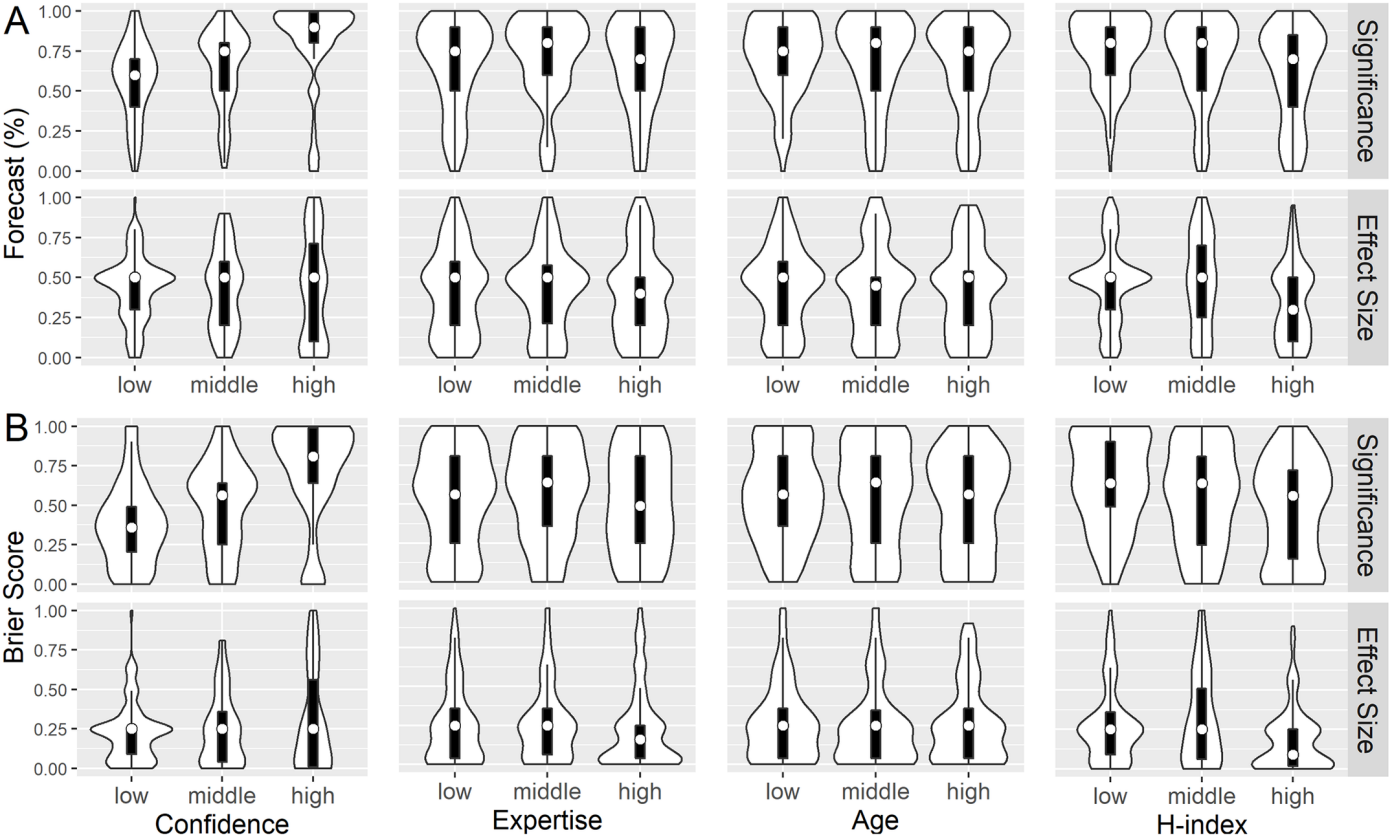
Forecasts and Brier scores by discretized predictors. All graphs show violin plots with embedded box plots and medians. Panels A and B display forecasts and Brier scores, respectively. Top and bottom rows show results for significance and effect-size forecasting, respectively. The columns show 4 predictors: self-reported confidence, self-reported expertise, age, and h-index (i.e., mean indices obtained from Scopus and Web of Science). Predictors were trichotomized to give approximately equal group sizes.

We also explored the relationship between 4 expert/forecast characteristics and skill scores: confidence in their forecasts, expertise, age, and h-index (the former 3 were self-reported; see Fig 3). The most notable impacts on the forecasts were confidence (r = 0.20, *p* < 0.01) and h-index (r = −0.15, *p* < 0.01). High confidence was indicative of poor accuracy, since mean Brier scores increased (r = 0.36, p < 0.01 for significance forecasts) and specificity decreased as confidence increased for both outcomes. For effect sizes, low confidence was associated with greater fifty-fifty forecasting, whereas high confidence led to greater variability in the forecasts. Specificity and accuracy improved with h-index for both outcome types. For significance, higher h-indices were associated with pessimistic, and thus more accurate, forecasts (r = −0.18, *p* < 0.01). For effect sizes, a low h-index was associated with a high degree of fifty-fifty forecasting, and a high h-index was associated with lower forecasts (r = −0.15, *p* < 0.01). In fact, experts with a high h-index did better than “fence-sitting,” with a mean Brier score of 0.17 and specificity of 0.81. When comparing expertise measures, experts were more accurate when they had general, compared to identified, expertise across h-index levels. The third of experts with the lowest h-indices were more discrepant, as Brier scores decreased by 0.09 on average when they had identified expertise, whereas the decrease was only 0.03 for the other two-thirds of experts.

Finally, we explored whether gender showed a relationship with forecasts and accuracy. Female experts gave forecasts that were about 8% higher on average than their male counterparts for both significance and effect size; female novices’ forecasts were about 6% higher on average than males for both. As a result, females’ Brier scores were higher than males’ both on replicability of significance (mean Brier score = 0.48, 95% CI (0.42 to 0.66) versus mean Brier score = 0.42, 95% CI [0.35 to 0.47]) and effect size (mean Brier score = 0.32, 95% CI [0.21 to 0.46] versus mean Brier score = 0.25, 95% CI [0.20 to 0.30]). Novice females also were less accurate than their male counterparts on significance replicability (mean Brier score = 0.56, 95% CI [0.50 to 0.67] versus mean Brier score = 0.46, 95% CI [0.41 to 0.66]) and effect size (mean Brier score = 0.28, 95% CI [0.21 to 0.39]) versus mean Brier score = 0.22, 95% CI [0.16 to 0.34]). The gender difference for replicability of effect-size forecasts was significant for both experts (0.07, 95% CI [−0.24 to −0.02]) and novices (0.06, 95% CI [−0.18 to −0.07]).

### Sensitivity analysis

The results from Study 29 have not yet been revealed. We performed a sensitivity analysis to determine how the results would change contingent on the eventual results of that study. The sensitivity results are shown in Figs 2 and 3 and S5 Table in the Supporting Information. Significance forecasts for Study 29 were less optimistic (median of 75%), and effect size forecasts were more pessimistic (median of 40%) than all but 1 of the other studies.

Overall, Brier scores will improve regardless of whether Study 29 reproduces the original effect. However, potential gains would be modest and would still underperform a fence-sitting strategy (Brier score = 0.25): If RP:CB results replicate Study 29, the overall mean Brier score would improve from 0.41 to 0.38, whereas if RP:CB does not replicate Study 29, it would be 0.40. If RP:CB replicates the results of Study 29, the mean Brier score for significance forecasts would improve significantly from a mean of 0.57 to 0.48 (95% CI [0.45 to 0.51]) and would show less improvement if Study 29 does not replicate, to 0.54 (95% CI [0.50 to 0.58]). The change for effect size forecasts would be smaller, but a positive replication would make the mean Brier score worse, from 0.26 to 0.29 (95% CI [0.26 to 0.32]), and a nonreplication would improve it to as good as a fence-sitting strategy (0.25, 95% CI [0.18 to 0.25]). All of the changes would be significantly different from the current results.

## Discussion

Our findings suggest that, on average, preclinical cancer researchers are overly optimistic in their beliefs about reproducibility. Half of all expert forecasts indicated that the replications were more likely to replicate original results than not, whereas only one-third indicated greater likelihood of replication failure. Overconfidence in reproducibility of specific results was particularly prevalent in significance forecasts, where 73% of expert forecasts favored successful replications. Forecasters were less certain (and more pessimistic) about replicating effect sizes.

There are several ways of interpreting our findings. Perhaps the most direct interpretation is that researchers consistently overestimated the validity of original reports. The high degree of optimism about statistical significance might reflect that experts believe effects in original studies are “real” (hence, they believe the replication results will also be positive and significant) but question the magnitude of treatment effects (hence, they show less optimism about effect sizes). Researchers may interpret the original significance levels as strong evidence that the effects are true positives, while they are more open to the possibility of type M (magnitude) error—which should be expected when samples sizes are small and/or measurements are noisy [22]. This interpretation implies researchers believe published results tend to be qualitatively valid but consistently overestimate effect sizes.

A second interpretation of our findings is that researchers were overly optimistic about the ease with which the original methods could be accurately replicated by independent laboratories or, more specifically, about the ability of these replicating laboratories to perform these particular replication studies [23]. RP:CB authors have stated that obtaining original protocols and materials was challenging, and originating labs often had to reconstruct materials that were unavailable [24]. Since publication of the first RP:CB studies, there has been a vigorous discussion about whether replication attempts were faithful to original methods, especially since 2 of the replication studies were deemed uninterpretable [25]. The RP:CB methods have been criticized for being too rigidly constrained by their registered protocols, preventing adjustment to challenges that arose while implementing original protocols [24–26]. Xenograft studies—which were the focus of our forecasting exercise—have come under particular criticism [25]. The 2 RP:CB replication studies that have received the most methodological criticisms had the second and third highest mean forecasts of significance. This suggests experts could have been overly optimistic about the challenge of executing direct replications in living biological systems or about the specialized skills RP:CB researchers would need to implement novel techniques.

A third interpretation is that forecasters were wrong in the specifics but right in the generalities. It is possible in each case that the original study was an overestimate of the effect size and the replication was an underestimate. As a consequence, further effort at replication might ultimately vindicate the experiments that did not successfully recapitulate effects observed in the original study. However, it is unlikely we would see the same pattern with such stark contrasts for every study. A different version of this interpretation is that some participants responded to our forecast questions with their beliefs about the underlying biological claims, rather than instantiations of them in particular laboratory experiments. In response, we point out that 2 of the 5 studies in our survey (19 and 21) were widely reported as having confirmed underlying biological claims, while 1 of the studies (15) was reported as having been disconfirmed [25]. If forecasts were right in the generalities, we would expect forecasts to be consistently higher for 19 and 21 than for 15 for both measures of replication. As Fig 1 shows, forecasts were pessimistic for Study 21 and relatively moderate for Study 15.

Although our study was not designed to discriminate between these various interpretations, it raises important questions about the way expert researchers interpret the sorts of high-impact findings tested in RP:CB. We find that biomedical scientists are inclined to overestimate the validity of original reports and/or underestimate the difficulty in conducting direct replications. The latter would perhaps be of less concern for science policy than the former. However, inability to appreciate the sensitivity of original reports to subtle laboratory conditions or aleatory uncertainty can result in potentially unfruitful debates about reproducibility, as well as wasted efforts as laboratories vainly attempt to follow protocols in original reports. It is beyond the scope of this paper to discuss the quality and reliability of experiments employed by the RP:CB. Scientific reports and news coverage of RP:CB have highlighted the difficulties in implementing replication studies (e.g., the commentaries reacting to the RP:CB results in *eLife*, *Nature*, and *Science*). We make no claim here about whether or not the RP:CB studies we examined constitute a gold standard of replication. We merely note that the manner in which such questions are resolved has implications for the interpretation of our results. Nevertheless, if—as many critics suggest—RP:CB experiments inadequately implemented original methods, our findings do not suggest these failings were apparent to our participants.

If forecasting improved with expertise, then some risks of overconfidence might be mitigated. Elsewhere, experts have been found to be as overconfident as novices, and the relationship between expertise and predictive skill is complex and task dependent [27,28]. In our study, greater topic expertise did not improve forecasting: indeed, experts displayed greater overconfidence when they were more familiar with a study and in general did not outperform our sample of trainees. This might be explained by experts having a tendency to take the “inside view”—a focus on the unique details of each specific experiment—over the “outside view”—a focus on the class of similar events [29]. The outside view tends to beat the inside view in predictive judgments [30]. On the other hand, our observations hint at the prospect that greater researcher impact (as measured by h-index) was associated with more accurate forecasts. Some reasons to expect experts to outperform nonexperts are they search for fewer total but more diagnostic cues and rely more on their existing knowledge and strategies [31].

We urge caution interpreting our results since reproducibility is difficult to define and measure [32]. Our survey scored forecasts using common forecasting methods that dichotomize whether studies reproduce based on 2 different criteria (significance and effect size). A strength of this method is that it creates predictive judgments that are verifiable. Participants’ judgments do not represent participants’ comprehensive beliefs about the treatment mechanisms at hand. However, it is rarely possible to capture such judgments in a manner that can be verified in a timely fashion. We do not suggest the forecasts collected in our study reflect judgments concerning fundamental scientific relationships generalized beyond the context in which they are tested. Additionally, replication is not an “all or nothing” phenomenon; 2 replication studies failed to reproduce statistical significance and had greatly diminished effect sizes but nevertheless showed effects that—when meta-analyzed with the original report—excluded the null hypothesis. Therefore, interpreting the overall success of these replications is to some degree in the eye of beholder. Moreover, a failed replication is not the same as evidence that the effect does not exist. However, there is no prescribed degree of disparity from an original result that is considered tolerable [33]. If the original and replication studies were sound methodologically, it can be difficult to judge if the original study overestimated or if the replication underestimated a true effect [34].

Our findings are subject to several limitations. First, despite a vigorous recruitment effort, our response rate was probably low, and our bounce rate was high. This may reflect some controversy surrounding the RP:CB. Nevertheless, we were able to capture a meaningful sample of experts: 138; this figure compares favorably with other studies requiring esoteric, expert research participants. The mean h-index for responders was somewhat lower than for nonresponders, suggesting our participants may have been slightly younger or marginally less productive than our sample population. However, the upper third of our sample had a high h-index (49.1 on average compared to 32.4 for the average nonresponder), including 22 experts with h-indices exceeding 40. Second, the gender imbalance in our expert sample is notably larger than in our novice sample. This imbalance may reflect an inherent gender imbalance between senior and junior researchers. Experts in life sciences tend to be primarily male (for example, 25% of tenured faculty positions in the biological and life sciences were held by females in 2013 [35]), while trainees tend be more female (for example, 53% of biological doctorates were awarded to women that year [36]). Females’ forecasts were about 6% higher on average, but the overall patterns of optimism held across gender. Third, our sample of experiments was small, and there was no variability in the reported results. We selected xenograft experiments because they are generally regarded as more predictive of clinical utility and the results were verifiable; this choice limited the number of available studies. We selected experiments a priori without any knowledge of outcomes, and the lack of variability in outcomes ultimately limited our ability to test researchers’ capacity to discriminate between studies that reproduce from those that do not. Our findings should therefore be replicated in a larger sample of experiments before drawing conclusions about the forecasting skill of cancer biology experts. Fourth, we do not know what the forecasters were intending to communicate when they offered forecasts. Forecast methods do not account for forecaster motivation. Indeed, the use of a “proper scoring rule” is intended to incentivize truthful—as opposed to strategic or motivated—disclosure of belief. Nevertheless, forecasters could have intended optimistic forecasts as a way of portraying the field positively or defending it against skepticism. Paradoxically, participants may have intended to express relative pessimism about reproducibility, since forecasts tended to be lower than participants’ expectations about perfectly reproducible effects (see S3 Fig in the Supporting Information). Such pessimistic forecasts could be a hedge so that the cancer field in general would appear better than predicted if replication results were “positive.” In any event, unconscious motives often play a role in survey responses and cannot be excluded as an explanation for the observed patterns. Fifth, our surveys assumed a certain amount of statistical knowledge. For example, asking individuals whether replication studies would achieve statistical significance (*p* ≤ 0.05) requires understanding what a *p*-value measures. We regard this as a potential strength, however. Scientific reports routinely convey claims through metrics like *p*-values or standardized mean differences. Therefore, the ability to derive valid inferences from such metrics is integral to forming judgments about effects and reproducibility.

Expert predictive judgment in biomedical research has not, to our knowledge, been previously studied. Scientists tend to focus on finding physical truths, and discrepancies between results often trigger debate about the methods used in each study. We suggest the role of scientific judgment is also important because physical truths can never be proven with certainty. The judgment of experts thus determines—at least in the short term—the way discrepant results are interpreted and which findings are taken as fact. Our study provides evidence that community-wide judgments about reproducibility are overoptimistic and overconfident.

The assessment and cultivation of researcher judgments, we suggest, may lead to a more efficient research enterprise. Knowing how well biomedical researchers can predict experimental outcomes is crucial for maintaining research systems that set appropriate research priorities, that self-correct, and that incorporate valid evidence into policy making. Regardless of how one interprets our results, these findings have potential policy implications, since they suggest that biomedical scientists have a difficult time anticipating outcomes when high-impact experiments are repeated. We believe it would be valuable to work to improve the judgments of researchers and trainees. There are common traits of domains in which experts’ forecasts tend to be more accurate and discriminating. Judgments must be formally quantified, and feedback should be timely, as unambiguous as possible, and easily compared to the original forecast [37]. Forecasting skill may also be better in domains in which expert judges are directly accountable to skeptical constituencies for the quality of their predictions [38]. We cannot expect researcher judgments to improve until the proper structure is in place to help them succeed.

## Methods

### Ethics statement

Human participant procedures were approved by the McGill University Faculty of Medicine Institutional Review Board (Protocol #A01-B05-16A). Each participant provided informed consent before commencing.

### Participants

We recruited experts using a 2-pronged search for authors of publications related to the first 6 studies in RP:CP (a flow chart of the sampling process is shown in S1 Fig in the Supporting Information). First, we used SCOPUS to identify all papers citing each original study. Second, we performed a “similar articles” search of each original study using PubMed. Both searches were conducted between February and July 2015. We captured the identity of all authors of the obtained articles. Individuals were eligible as experts if (a) they were named as a corresponding author on any article captured in our 2 searches or (b) their name appeared 3 times, in any author position, in 1 of the searches. Eligible experts were sent up to 5 email invitations spaced at least 1 week apart or until an invitee completed the survey or actively declined.

As a comparator, we also created a sample of biomedicine novices by circulating survey invitations to all graduate and postdoctoral students in programs with relevant cancer training at 2 leading research universities—1 in eastern Canada and 1 in the northeast United States. Use of such a comparator was prompted by the observation that experts in other fields sometimes underperform nonexperts when making predictive judgments [39,40]. We incentivized invitees by offering a C$200 reward to the top 2 forecasters and C$50 to the 2 next best forecasters. We also offered the top 3 forecasters the option of having their names published at http://www.translationalethics.com/forecast-scoreboard/.

A total of 2,667 experts were invited to participate: 12.3% (329) of invitations bounced, and 1.57% (42) actively declined. Assuming that all nonbounced invitations sent were actually received by our targets, 6% (138) of invited experts forecasted at least 1 study. Because novice invites were circulated by graduate program directors, we cannot calculate response rates for novices. We excluded the data of 1 participant who completed the survey well below our threshold of 7 minutes. We disqualified 12 forecasts for which participants declared knowledge of the replication results.

### Surveys

We based our queries of reproducibility on mouse tumor growth curves in the first 6 studies planned by RP:CB. We used mouse xenograft experiments because they are believed by many to be the most prognostic of clinical utility. Ideally, we would solicit subjective probabilities (i.e., forecasts) on whether whole studies reproduced. However, every study contained multiple experiments, and the definition of “reproduction” is itself highly debated [32,33]. As a result, using such an approach would make it impossible to verify the accuracy of forecasts. Instead, we used an approach that minimized the interpretational ambiguity of our questions and the ensuing forecast data. Each experiment had a single effect (treatment versus control) that could be said to represent the main finding of the study. This allowed us to collect predictive judgments for each study and to create objective and unambiguous methods for scoring each prediction. To maintain a mean survey duration of 30 minutes, we created 2 batches of surveys, each asking about 3 RP:CB studies. Survey questions were reviewed and approved by cancer preclinical researcher consultants.

For each replication forecast, we gave participants the study title, authors, and links to both the original study and the registered report for the replication. We also gave them the relevant figure from the original paper, with annotations indicating the effect our query centered on. Participants were asked to indicate their subjective probabilities (in percent chance format) that (a) the replication experiments would “also be statistically significant in the same direction (as the original study)” and (b) the effect size of the replication would be “as large or larger than the original effect size.” We chose these particular questions over more complicated judgments to minimize the degree to which our findings would be confounded by participants’ statistical sophistication [7,32,41].

For secondary analyses, we probed (a) confidence in each forecast, (b) self-rated expertise on each experiment, (c) additional cognitive variables shown to correlate with forecast skill in other domains: actively openminded thinking [42] and belief in the progress of medical research-adapted from an economic boomster-doomster scale [43], and (d) questions about their familiarity with and the anticipated influence of the RP:CB on how they read papers and design studies. We also asked participants to estimate the overall replication rate the RP:CB will report, but we do not present this data in the current paper since it is premature to do so, and we do not want to confuse researchers’ beliefs about xenograft studies with their beliefs about in vitro studies. Participants were also asked the following: “Imagine a replication study perfectly reproduces the effects observed in an original study. How likely would the effect be as large or larger than that provided in the original study?” We provided an open text screen for respondents to provide thoughts and feedback on our survey. Further details, including the study protocol, are provided in the Supporting Information (see S1 Text). Surveys were conducted online using Surveymonkey. The order of studies in each survey was drawn randomly for each participant.

We analyzed the results of the first 5 studies whose outcomes are resolved and that have been published in *eLife* [17–21], and we present a sensitivity analysis of the sixth study by describing how the forecast results would change if it replicated the results or not. Accuracy of forecasts were calculated using the Brier score, a proper scoring rule for forecast accuracy that measures the squared deviation between a forecast probability and the actual outcome (coded 0 for nonoccurrence and 1 for occurrence) [44,45], so a lower Brier score denotes a more accurate forecaster than a higher score. A forecaster who behaves like a clairvoyant, correctly classifying all outcomes with complete confidence, would get a perfect Brier score of 0. In contrast, a forecaster who invariably guesses 50%—a common response for participants who do not know how to answer questions about the probability of binary options, sometimes called “the fifty-fifty blip” [46]—would have a Brier score of 0.25. We report mean Brier scores with bias-corrected 95% bootstrap confidence intervals with 5,000 resamples. All hypothesis tests were conducted using bootstrap t-tests based on whether 95% bootstrap confidence intervals of the mean difference contained the value 0. We also report the extent to which forecasts correctly identified which studies would not replicate using specificity (i.e., the “true negative rate”), which is normalized by the sum of true negative and false positive forecasts. Data analysis was conducted using R statistical software.

## Supporting information

S1 Fig Flow chart of expert sampling process. (TIF)

S2 Fig Direct comparison of forecasts and Brier scores by rank for survey 1. The first row shows forecasts and the second shows brier scores. The first column shows significance and the second shows effect size. For each panel, the histogram is the aggregate as all of respondents, the red line is for experts and the blue line is for novices.
(TIF)

S3 Fig Likelihood of reproducing upon perfect replication. In the top row, the dark gray histograms show the forecast of a (genericized) perfect replication, and density lines show actual effect size forecasts. The bottom row is the perfect replication ratio: log(forecast/perfect replication). Thus, a negative value indicates the forecast is lower than the perfect replication. The left column shows data from all experts, whereas the right column shows survey 1 data comparing experts to novices. There are multiple interpretations of the idea of a perfect replication. 1) The modal response was .5, which may indicate that the original effect size is a reasonable estimate of the true effect; 2) some participants thought it should be high (.95 for students; 1 for novices), perhaps based on the possibility of Type I error from the original study; 3) some participants expected .8, perhaps based on the power and Type II error from the replication study; 4) and yet others thought it was less than 50% (20% or 30%), suggesting they may have a cynical view of reproducibility in this field. There is a strong peak in panels 3 and 4 for participants who made forecasts in line with a perfect replication. Beyond that, the trend is to make forecasts below a hypothetical perfect replication, so the forecasters were consistently acknowledging that real replication studies are flawed to some degree. Experts were more likely than novices to forecast higher than the perfect replication seemingly suggesting that some of these replication studies were highly likely to replicate the original results compared to other studies.
(TIF)

S1 Table Completions by survey. (DOCX)

S2 Table Forecast descriptive statistics and prediction accuracy by trichotomized predictors. (DOCX)

S3 Table Descriptive statistics of discretized predictors. (DOCX)

S4 Table Original and replication study results. (DOCX)

S5 Table Sensitivity analysis of Brier scores by outcome and identified expertise. (DOCX)

S1 Text Study protocol. (DOCX)

S1 Data Data file. Potentially identifying information has been redacted to protect the anonymity of the participants.
(CSV)

S2 Data Codebook. (XLSX)

## Abbreviations

FDA: Food and Drug Administration
RP:CB: Reproducibility Project: Cancer Biology
