# Supplementary figures and images for: Can cancer researchers accurately judge whether preclinical reports will reproduce?
Source: PLoS Biol. 2017 Jun 29;15(6):e2002212. doi: 10.1371/journal.pbio.2002212 (PMC5490935; doi:10.1371/journal.pbio.2002212)

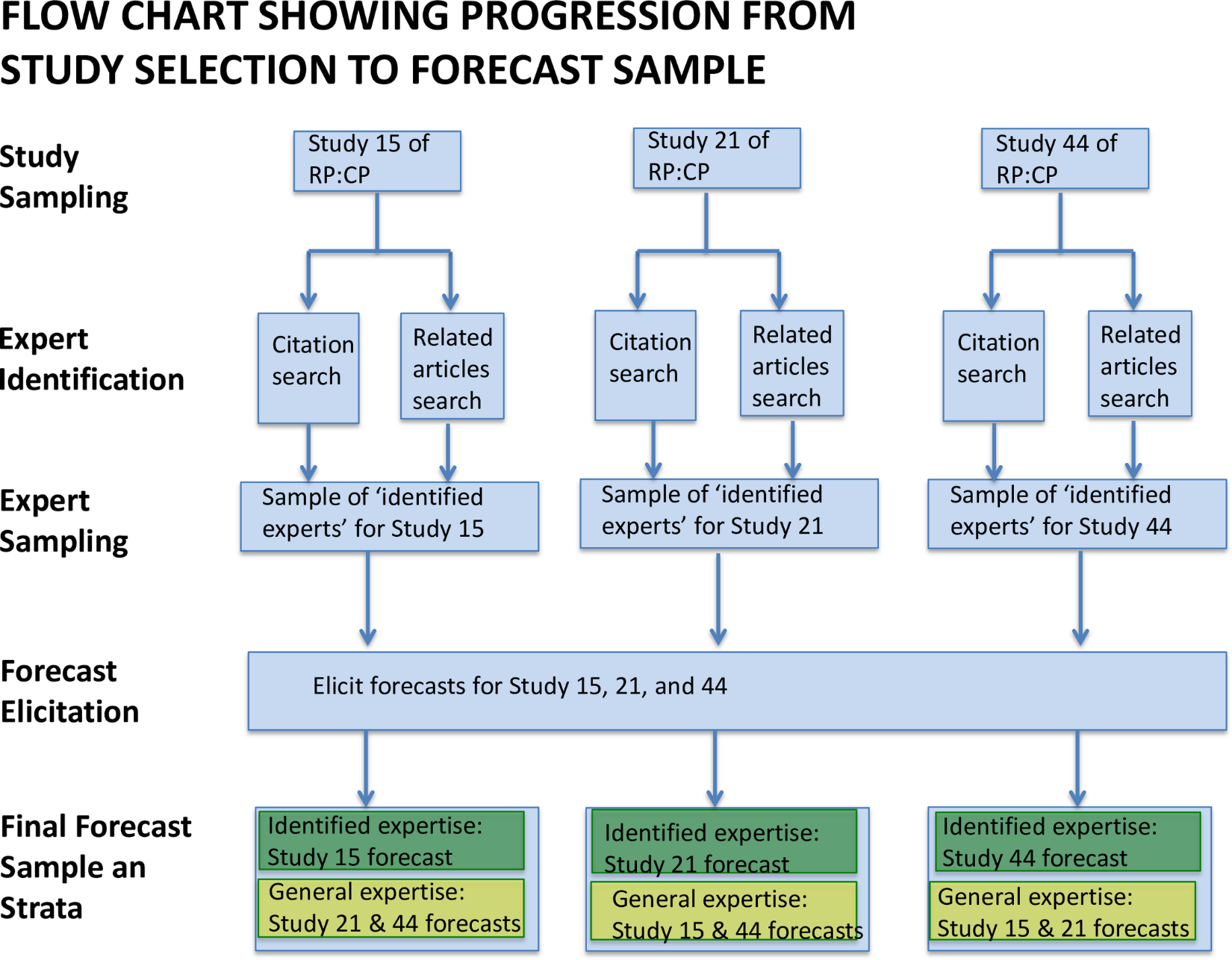

Supplement: S1 Fig — (TIF) [file pbio.2002212.s001.tif]

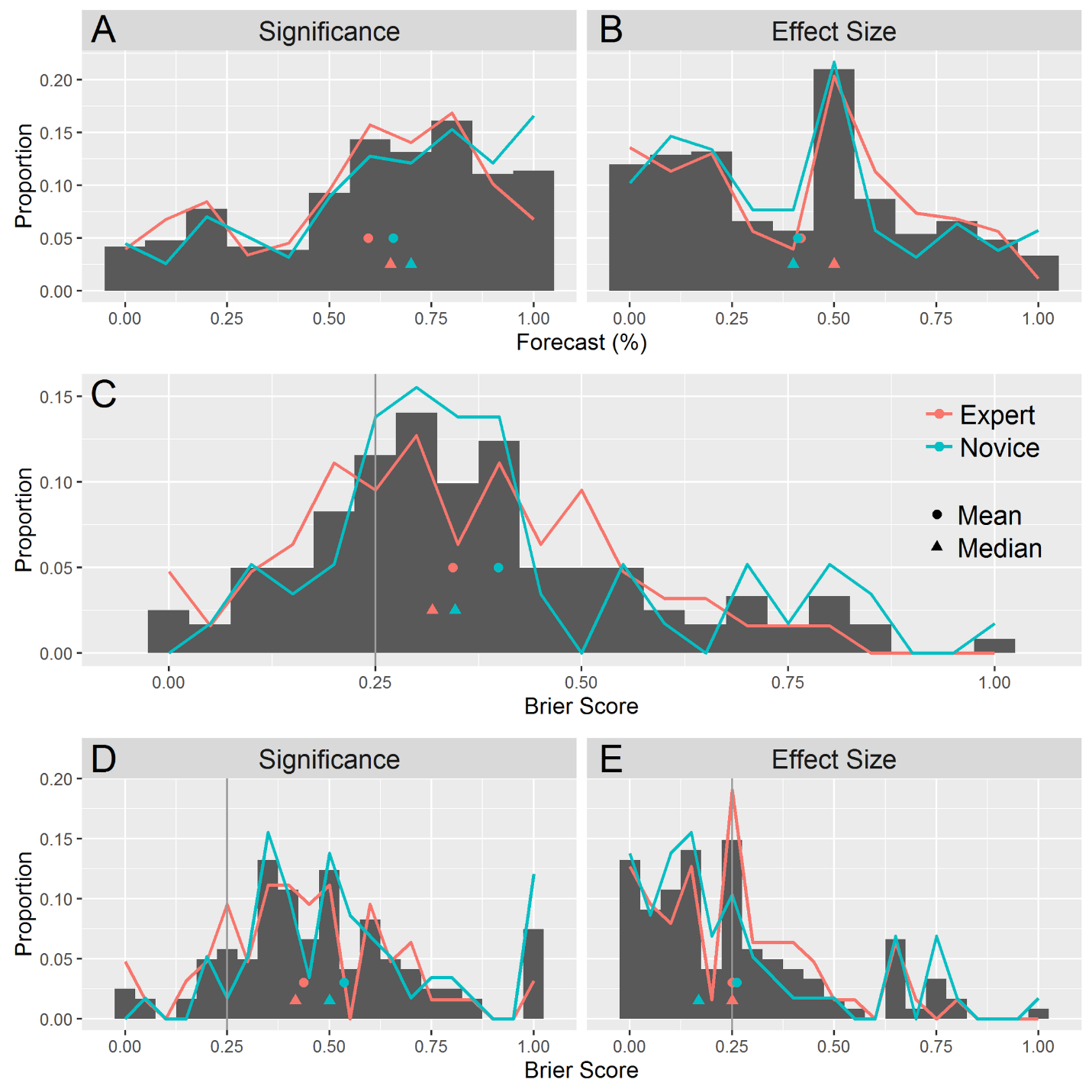

Supplement: S2 Fig — The first row shows forecasts and the second shows brier scores. The first column shows significance and the second shows effect size. For each panel, the histogram is the aggregate as all of respondents, the red line is for experts and the blue line is for novices. (TIF) [file pbio.2002212.s002.tif]

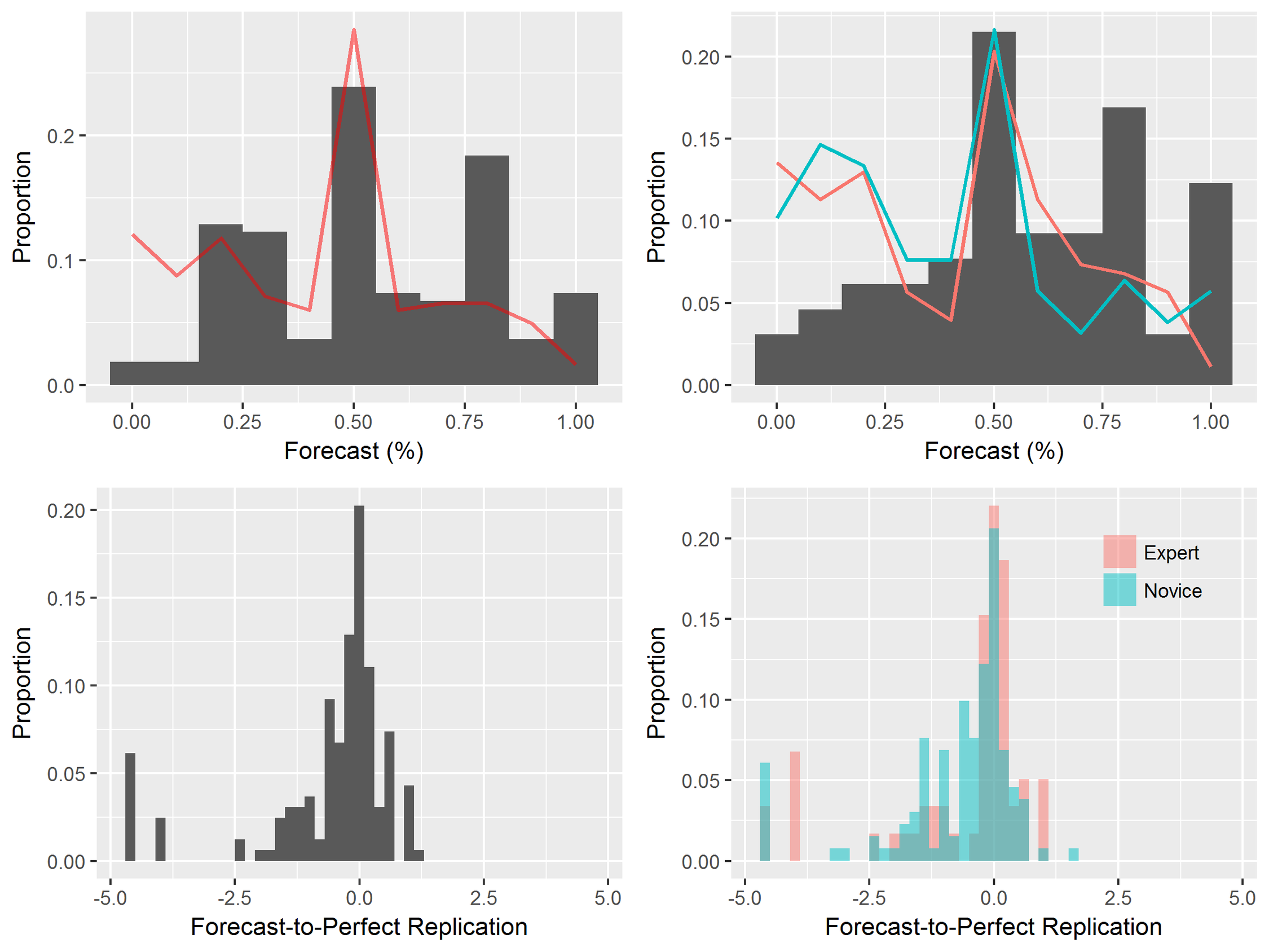

Supplement: S3 Fig — In the top row, the dark gray histograms show the forecast of a (genericized) perfect replication, and density lines show actual effect size forecasts. The bottom row is the perfect replication ratio: log(forecast/perfect replication). Thus, a negative value indicates the forecast is lower than the perfect replication. The left column shows data from all experts, whereas the right column shows survey 1 data comparing experts to novices. There are multiple interpretations of the idea of a perfect replication. 1) The modal response was .5, which may indicate that the original effect size is a reasonable estimate of the true effect; 2) some participants thought it should be high (.95 for students; 1 for novices), perhaps based on the possibility of Type I error from the original study; 3) some participants expected .8, perhaps based on the power and Type II error from the replication study; 4) and yet others thought it was less than 50% (20% or 30%), suggesting they may have a cynical view of reproducibility in this field. There is a strong peak in panels 3 and 4 for participants who made forecasts in line with a perfect replication. Beyond that, the trend is to make forecasts below a hypothetical perfect replication, so the forecasters were consistently acknowledging that real replication studies are flawed to some degree. Experts were more likely than novices to forecast higher than the perfect replication seemingly suggesting that some of these replication studies were highly likely to replicate the original results compared to other studies. (TIF) [file pbio.2002212.s003.tif]
