# Supplementary material for: Can cancer researchers accurately judge whether preclinical reports will reproduce?
Source: PLoS Biol. 2017 Jun 29;15(6):e2002212. doi: 10.1371/journal.pbio.2002212 (PMC5490935; doi:10.1371/journal.pbio.2002212)
Supplement: S1 Table — (DOCX) [file pbio.2002212.s004.docx]

|  |  | Complete | Partial | Total |
| --- | --- | --- | --- | --- |
| Total | | 162 | 34 | 196 |
|  |  |  |  |  |
| Experts | | 118 | 20 | 138 |
|  | Non-incentivized | 97 | 17 | 114 |
|  | Incentivized | 21 | 3 | 24 |
| Survey 1 | | 54 | 9 | 63 |
|  | Non-incentivized | 47 | 8 | 55 |
|  | Incentivized | 7 | 1 | 8 |
| Survey 2 | | 64 | 11 | 75 |
|  | Non-incentivized | 50 | 9 | 59 |
|  | Incentivized | 14 | 2 | 16 |
|  |  |  |  |  |
| Students (all Survey 1) | | 44 | 14 | 58 |
