# Supplementary material for: Can cancer researchers accurately judge whether preclinical reports will reproduce?
Source: PLoS Biol. 2017 Jun 29;15(6):e2002212. doi: 10.1371/journal.pbio.2002212 (PMC5490935; doi:10.1371/journal.pbio.2002212)
Supplement: S2 Table — (DOCX) [file pbio.2002212.s005.docx]

| Predictor | Outcome | Level | N | mean | SD | median | skew | min | max |
| --- | --- | --- | --- | --- | --- | --- | --- | --- | --- |
| Confidence |  | Low | 71 | 57.92 | 11.53 | 0.6 | -1.23 | 0.12 | 0.7 |
|  | Significance | Middle | 80 | 79.28 | 2.43 | 0.8 | -0.27 | 0.75 | 0.85 |
|  |  | High | 86 | 93.60 | 4.22 | 0.91 | 0.60 | 0.9 | 1 |
| Confidence |  | Low | 73 | 54.14 | 13.20 | 0.5 | -1.46 | 0.01 | 0.7 |
|  | Effect size | Middle | 69 | 79.30 | 2.15 | 0.8 | -0.84 | 0.75 | 0.85 |
|  |  | High | 70 | 93.81 | 4.44 | 0.9 | 0.50 | 0.9 | 1 |
| Expertise | All | Low | 77 | 2.10 | 0.82 | 2 | -0.16 | 1 | 3 |
|  |  | Middle | 90 | 4.44 | 0.44 | 4.42 | 0.27 | 4 | 5 |
|  |  | High | 67 | 6.42 | 0.47 | 6 | 0.29 | 6 | 7 |
| Age | All | Low | 41 | 34.59 | 4.55 | 35 | -1.02 | 20 | 40 |
|  |  | Middle | 47 | 45.38 | 3.12 | 45 | 0.15 | 41 | 50 |
|  |  | High | 46 | 59.65 | 7.10 | 58.5 | 1.19 | 51 | 81 |
| h-index | All | Low | 41 | 8.26 | 4.31 | 9.5 | -0.38 | 0 | 14.5 |
|  |  | Middle | 45 | 19.91 | 3.72 | 19 | 0.45 | 15 | 27 |
|  |  | High | 43 | 46.78 | 19.27 | 41 | 1.69 | 28 | 113 |
