# Supplementary material for: Can cancer researchers accurately judge whether preclinical reports will reproduce?
Source: PLoS Biol. 2017 Jun 29;15(6):e2002212. doi: 10.1371/journal.pbio.2002212 (PMC5490935; doi:10.1371/journal.pbio.2002212)
Supplement: S3 Table — (DOCX) [file pbio.2002212.s006.docx]

| Predictor | Outcome | Level | N | Mean | Median | SD | Skew | Mean Brier | Median Brier | Specificity |
| --- | --- | --- | --- | --- | --- | --- | --- | --- | --- | --- |
| Confidence | All | Low | 246 | 0.49 | 0.50 | 0.23 | -0.16 | 0.30 | 0.25 | 0.48 |
|  |  | Middle | 240 | 0.54 | 0.51 | 0.27 | -0.29 | 0.38 | 0.37 | 0.40 |
|  |  | High | 285 | 0.62 | 0.80 | 0.35 | -0.60 | 0.52 | 0.59 | 0.32 |
|  | Significance | Low | 111 | 0.57 | 0.60 | 0.24 | -0.45 | 0.38 | 0.41 | 0.31 |
|  |  | Middle | 135 | 0.43 | 0.50 | 0.20 | -0.15 | 0.50 | 0.56 | 0.21 |
|  |  | High | 125 | 0.65 | 0.75 | 0.24 | -0.83 | 0.71 | 0.81 | 0.12 |
|  | Effect Size | Low | 115 | 0.43 | 0.50 | 0.25 | 0.14 | 0.23 | 0.25 | 0.68 |
|  |  | Middle | 153 | 0.80 | 0.90 | 0.27 | -1.77 | 0.25 | 0.25 | 0.63 |
|  |  | High | 132 | 0.42 | 0.50 | 0.33 | 0.20 | 0.30 | 0.25 | 0.61 |
| Expertise | All | Low | 239 | 0.54 | 0.50 | 0.30 | -0.21 | 0.39 | 0.45 | 0.41 |
|  |  | Middle | 285 | 0.57 | 0.60 | 0.30 | -0.35 | 0.42 | 0.45 | 0.36 |
|  |  | High | 205 | 0.53 | 0.50 | 0.30 | -0.13 | 0.37 | 0.34 | 0.45 |
|  | Significance | Low | 120 | 0.67 | 0.75 | 0.27 | -0.75 | 0.53 | 0.64 | 0.24 |
|  |  | Middle | 119 | 0.42 | 0.50 | 0.27 | 0.15 | 0.58 | 0.64 | 0.14 |
|  |  | High | 143 | 0.71 | 0.80 | 0.26 | -1.04 | 0.51 | 0.49 | 0.23 |
|  | Effect Size | Low | 142 | 0.43 | 0.50 | 0.26 | 0.04 | 0.25 | 0.25 | 0.63 |
|  |  | Middle | 103 | 0.66 | 0.70 | 0.28 | -0.72 | 0.26 | 0.25 | 0.64 |
|  |  | High | 102 | 0.40 | 0.40 | 0.27 | 0.32 | 0.23 | 0.13 | 0.70 |
| Age | All | Low | 238 | 0.57 | 0.60 | 0.28 | -0.31 | 0.41 | 0.40 | 0.35 |
|  |  | Middle | 263 | 0.55 | 0.50 | 0.31 | -0.21 | 0.40 | 0.45 | 0.42 |
|  |  | High | 254 | 0.54 | 0.50 | 0.30 | -0.25 | 0.39 | 0.43 | 0.41 |
|  | Significance | Low | 119 | 0.71 | 0.75 | 0.24 | -0.76 | 0.56 | 0.56 | 0.14 |
|  |  | Middle | 119 | 0.44 | 0.50 | 0.25 | -0.01 | 0.55 | 0.58 | 0.23 |
|  |  | High | 133 | 0.68 | 0.80 | 0.28 | -0.84 | 0.51 | 0.60 | 0.23 |
|  | Effect Size | Low | 130 | 0.42 | 0.45 | 0.27 | 0.23 | 0.26 | 0.25 | 0.60 |
|  |  | Middle | 128 | 0.65 | 0.75 | 0.29 | -0.81 | 0.25 | 0.25 | 0.67 |
|  |  | High | 126 | 0.42 | 0.50 | 0.27 | 0.10 | 0.26 | 0.25 | 0.64 |
| h-index | All | Low | 239 | 0.61 | 0.60 | 0.27 | -0.35 | 0.46 | 0.49 | 0.30 |
|  |  | Middle | 250 | 0.59 | 0.60 | 0.30 | -0.45 | 0.44 | 0.43 | 0.34 |
|  |  | High | 242 | 0.47 | 0.50 | 0.30 | 0.01 | 0.32 | 0.33 | 0.52 |
|  | Significance | Low | 121 | 0.76 | 0.80 | 0.22 | -0.93 | 0.64 | 0.69 | 0.09 |
|  |  | Middle | 118 | 0.46 | 0.50 | 0.24 | 0.02 | 0.56 | 0.57 | 0.18 |
|  |  | High | 125 | 0.70 | 0.80 | 0.26 | -0.94 | 0.46 | 0.46 | 0.31 |
|  | Effect Size | Low | 125 | 0.47 | 0.50 | 0.29 | -0.05 | 0.27 | 0.25 | 0.61 |
|  |  | Middle | 121 | 0.60 | 0.70 | 0.30 | -0.61 | 0.32 | 0.26 | 0.53 |
|  |  | High | 121 | 0.34 | 0.30 | 0.24 | 0.32 | 0.18 | 0.15 | 0.80 |
