# Supplementary material for: Can cancer researchers accurately judge whether preclinical reports will reproduce?
Source: PLoS Biol. 2017 Jun 29;15(6):e2002212. doi: 10.1371/journal.pbio.2002212 (PMC5490935; doi:10.1371/journal.pbio.2002212)
Supplement: S4 Table — (DOCX) [file pbio.2002212.s007.docx]

|  | Significance | | | | Effect Size | | | | |
| --- | --- | --- | --- | --- | --- | --- | --- | --- | --- |
| Study | Original | Replication | z | SE | Original | Replication | SMD | SD | Unit |
| 15 | 0.05 | 0.826 | 4.61 | 0.17 | 1.61 | 0.22 | 0.58 | 2.39 | Cohen's d |
| 21 | 0.001 | 0.105 | 1.15 | 0.09 | 1.36 | 0.93 | 0.19 | 2.28 | Glass' Δ |
| 44 | 0.006 | 0.99 | 6.3 | 0.16 | 1.91 | -0.21 | 0.72 | 2.93 | log(HR) |
| 19 | 0.0002 | 0.205 | 2.08 | 0.1 | 2.3 | 0.56 | 0.69 | 2.53 | Cohen's d |
| 29 | 0.017 | 0.536 | 2.82 | 0.18 | 2.33 | 0.36 | 1.28 | 1.53 | log(HR) |
| 39 | 0.0005 | 0.104 | 1.04 | 0.1 | 2.54 | -1.13 | 1.28 | 2.88 | Glass' Δ |
