# Supplementary material for: Can cancer researchers accurately judge whether preclinical reports will reproduce?
Source: PLoS Biol. 2017 Jun 29;15(6):e2002212. doi: 10.1371/journal.pbio.2002212 (PMC5490935; doi:10.1371/journal.pbio.2002212)
Supplement: S5 Table — (DOCX) [file pbio.2002212.s008.docx]

|  |  |  | Mean Brier | Bootstrap C.I. | | Median Brier |  |  |  |
| --- | --- | --- | --- | --- | --- | --- | --- | --- | --- |
| Study 29 | Outcome | Expert |  | Lower | Upper |  |  | Specificity | Sensitivity |
| Unknown | All | All | 0.41 | 0.38 | 0.45 | 0.42 |  | 0.38 |  |
| Succeed |  |  | 0.38 | 0.36 | 0.41 | 0.40 |  |  | 0.54 |
| Fail |  |  | 0.40 | 0.37 | 0.43 | 0.40 |  | 0.39 |  |
| Unknown | Significance | All | 0.57 | 0.52 | 0.61 | 0.58 |  | 0.19 |  |
| Succeed |  |  | 0.48 | 0.45 | 0.51 | 0.49 |  |  | 0.78 |
| Fail |  |  | 0.54 | 0.50 | 0.58 | 0.54 |  | 0.2 |  |
| Unknown | Effect size | All | 0.26 | 0.22 | 0.30 | 0.25 |  | 0.61 |  |
| Succeed |  |  | 0.29 | 0.26 | 0.32 | 0.26 |  |  | 0.23 |
| Fail |  |  | 0.25 | 0.22 | 0.29 | 0.22 |  | 0.64 |  |
| Unknown | All | Identified | 0.44 | 0.39 | 0.48 | 0.45 |  | 0.32 |  |
| Succeed |  |  | 0.40 | 0.36 | 0.43 | 0.40 |  |  | 0.59 |
| Fail |  |  | 0.43 | 0.39 | 0.47 | 0.43 |  | 0.35 |  |
| Unknown | All | Unidentified | 0.39 | 0.35 | 0.42 | 0.41 |  | 0.41 |  |
| Succeed |  |  | 0.38 | 0.35 | 0.41 | 0.37 |  |  | 0.47 |
| Fail |  |  | 0.38 | 0.34 | 0.42 | 0.39 |  | 0.42 |  |
