# Supplementary material for: Can cancer researchers accurately judge whether preclinical reports will reproduce?
Source: PLoS Biol. 2017 Jun 29;15(6):e2002212. doi: 10.1371/journal.pbio.2002212 (PMC5490935; doi:10.1371/journal.pbio.2002212)
Supplement: S1 Text — (DOCX) [file pbio.2002212.s009.docx]

**Appendix
Study Protocol**

| TITLE  **Can Cancer Experts Accurately Forecast the Replication of Cancer Biology Trials?** |
| --- |
| INTRODUCTION  The replicability of experimental findings is a key tenant of science. There is mounting evidence that many findings in the basic and preclinical literatures cannot be reproduced.[1–4] This has important policy and ethical implications - particularly in the context where non-reproducible findings are used to launch clinical trials, costly research initiatives, or support policy.  To date, most investigations of reproducibility have used one of two different methods. First, meta-analytic tools have been used to provide evidence that effect sizes are overestimated, that there is an excess of statistically significant findings, or that effects between related studies are not concordant with each other.[5,6] A second method has involved direct and strict replication of experimental findings in order to gauge the degree to which findings are reproducible.[4] This latter approach has been used in spinal cord injury, cancer, and most recently, psychology. Together, these two streams of evidence tell us that many published reports, in diverse research areas, describe effects that are exaggerated, or in some cases, spurious.  An important limitation of this literature, however, is its focus on the experiment and its effect, rather than the beliefs they produce. Experiments are merely the substrate out of which scientific belief is produced. Experiments are conducted not for their own sake, but rather to support valid inferences about natural phenomena. Phenomena cannot be proven by individual results; instead theories are developed based on the accumulation of evidence. Non-reproducibility threatens valid inference from a scientific, policy and ethical standpoint. To our knowledge, no one has yet looked at the validity of inferences that experts make about the reliability of research findings in basic science or biomedical research.  This study will use forecast methodologies to investigate the accuracy of researchers’ judgments about the reproducibility of findings in cancer biology. In particular, we will investigate whether researchers have an accurate understanding of the replicability rate in their field, and if they have the capacity to discriminate between effects that can be confirmed and false positive results. We will also investigate whether there are certain types of individuals who are better at discriminating reproducible from unreproducible findings. By collecting forecasts from a large sample of researchers, we can also assess whether the weight of opinion in research communities is swayed - or resistant to - potentially exaggerated effects reported in studies. By investigating the inferential dimension of the reproducibility crisis, the findings of this investigation will clarify whether scientists (and the community) are savvy consumers of biomedical evidence.  Quantifying beliefs about replicability rates will have important implications for scientific methodology and policy. Results will fall between four extremes. 1) Experts accurately predict high replication rates indicating the scientific method is working and published results are high quality. 2) Experts believe replication rates are high, but they are low indicating researchers are overly trusting of published findings. 3) Experts believe replication rates are low, but they are high indicating the recent scrutiny about research methodology and/or stream of bad press sometimes describing sciences as being in “crisis” is resulting in seepage the scientific community (see [7] for an analysis of the impact of seepage on climate change research). 4) Experts accurately predict low replication rates, indicating the research community has good savvy regarding the interpretation of research findings, but researchers are exercising enough flexibility in scientific methodology to lead to an excess of significant results.  This investigation will piggyback on a project underway in cancer biology that is reproducing 50 recent^[[1]](#footnote-1)^, landmark papers in cancer biology.[8] We will collect a series of subjective probability forecasts of dichotomous outcomes from a large sample of cancer researchers on each of the experiments underway in the cancer reproducibility initiative (e.g. “what is your subjective probability that the effect size measured in the replication of experiment 5 will be as large or larger than the original effect size?”) We can then score individual researchers on their ability to discern reproducible from non-reproducible finding. We will also score the broader community, and probe whether researchers of the original reports, or possessing certain cognitive traits, are better or worse at anticipating the reproducibility of their studies.  This project is a module that fits within the parameters of our originally funded grant. We have maintained several concurrent modules testing forecast skill in basic and clinical research - all have received IRB approval. |
| PRIMARY OBJECTIVES  To determine how well the expert cancer research community, in the aggregate, can forecast the overall replication rate of high impact preclinical cancer studies and the reproducibility of specific high impact basic and preclinical cancer experiments.  To determine how well individual experts in this community can forecast the overall replication rate of high impact preclinical cancer studies and the reproducibility of specific high impact basic and preclinical cancer experiments. |
| SECONDARY OBJECTIVES   1. What are the cognitive characteristics of the experts who update more appropriately? 2. Determine whether beliefs about the pace of progress in medical research (AKA medical boomster / doomster scale score) correlates with forecast skill. 3. Determine demographic characteristics of better forecasters. 4. Determine target study features (in vivo vs. in vitro, etc) with higher forecast success rates 5. How well do forecasters update after seeing results? 6. What are the demographic characteristics of experts who update more appropriately? 7. What types of evidence do the most effective forecasters prioritize, and what study features are easiest to predict? 8. Combine several above results to identify which trial and forecaster characteristics lead to more accurate forecasts. 9. Determine whether researchers who originate a report are more or less accurate than independent experts, individuals associated with the team replicating the experiment and non-experts. |
| METHOD OVERVIEW  We will use both ‘binary’ forecast methodologies (i.e. subjective probability on binary outcomes) to collect forecasts for individual replication studies, and ‘simplified continuous’ elicitations on select studies. When replication studies close, we will collect study outcomes, determine forecast scores using a proper scoring rule (called Brier score), and collect updated forecasts. |
| SAMPLING  Study sampling and sample sizes:  Our study sampling frame will consist of all (approximately 35) studies being tested in the Reproducibility Project:Cancer Biology. We will categorize the studies based on theoretical and practical features. We will remove studies due to lack of feasibility (e.g. if there is not a clear relevant effect to predict, such as many in vitro studies especially those focused on cell signaling; if maturation date is too far in the future). We will prioritize studies whose results will be published sooner.  We will work with an independent expert to create or edit two to three questions for each study that can be rendered into dichotomized forecasts. In stage 1, participants will forecast all of the first six (all based on mouse models) studies from the cancer reproducibility project plans to publish. Participants will answer two binary elicitations for six events and simplified continuous elicitations for two events.  When mouse survival or tumor growth curves are used, questions will be created around them. When there are no mouse studies, we will shape a question around a study with a quantitative outcome. We plan to conduct a brief pilot to ensure the question types are relatively intuitive. Question formats might vary slightly depending on pilot results.  For the binary elicitation, our questions will take the two^[[2]](#footnote-2)^ of the following general forms:   1. How likely is it that the replication also be significant (at p ≤ .05)? 2. How likely is it that the effect size of the replication will be as large or larger than the original effect size?   For the simplified continuous elicitation (SCE) for the effect size of the specified treatment group, we will use a four question format, based on [9]:  1. What is the lowest effect size you realistically anticipate will be reported for study X?  2. What is the highest effect size you realistically anticipate will be reported for study X?  3. What is your best guess of the effect size you realistically anticipate will be reported for study X?  4. How confident are you (from 0% to 100%) that your estimated interval (lowest to highest) will include the reported effect size from study X?  Investigator sampling and planned strata:  All respondents will be at least 18 years old and will complete an informed consent.  Our primary sample will include two strata:  *a) Independent Experts*: we will create a sample of about 50 to 100 eligible independent international experts by compiling a list of authors from two searchers based on each original study undergoing replication: a) *related article* searches on PubMed and b) articles citing the original report. We will include corresponding authors and anyone who appears at least three times in any single search.  b) Novices: we will recruit a sample of about 50 to 100 medical undergraduate or upper level undergraduate biology majors, graduate students, or postdoctoral fellows at various universities via department mailing lists to participate in our study.  We will aim to include two secondary strata^[[3]](#footnote-3)^:  *c) Originators*: we will contact each co-author on each original research publication for an elicitation.  *d) Replicators*: we will contact each co-author on each replication protocol - only in cases where data collection has not yet been initiated.  Subjects will be approached through email (except non-experts, who may be approached in a class), and administered the questionnaire electronically.  Individuals will be approached up to five times before declaring them as having declined participation.  Sample size:  Our target sample size is as follows:  We will collect forecasts on 3 events in a half hour. We will collect 2 binary forecasts on 6 effects from 6 experiments + simplified continuous forecasts on overall replication rates). We will split the trial sample into two surveys to minimize the number of trials for which participants only have general expertise.  We will target 30 forecasts per participant on 6 events.  We will aim for 150 participants (50X2=100 independent experts + 50 novices) and expect a minimum of 90 (30X2=60 independent experts + 30 novices). |
| INTERVIEW AND DATA COLLECTION  Interviews will be conducted in 2 stages:   1. Intake interview: in one 30-minute survey presented over the internet (or in person, or by paper and pencil), we will present subjects with a one-page summary sheet describing each replication study (see Appendix 2), as well as a questionnaire (see Appendix 3). Individuals will be approached using appropriate elicitation technique, whereby we first a) describe the goals of the project and how to complete the questionnaire; b) attempt to motivate good performance by explaining how scoring works; c) provide a simple warm up question (asking for a weather forecast); d) elicit replication forecasts; and e) collect additional cognitive and attitudinal measures. The questionnaire will first collect forecasts, then individual difference variables (as applicable), then demographic variables. 2. Forecasts: subjective probabilities on target experiments.    - 1. 2 binary: Significant difference and direction on 3 replication studies      2. 2 @ 4 parameter simplified continuous (best estimate, range, confidence) on overall replication rates.    1. Individual difference variables       1. Actively Open-Minded Thinking[10]       2. Pace of progress of medical research (AKA Medical Boomster-Doomster, adapted from [11])       3. Self-report of expertise for each study       4. Confidence rating of each forecast       5. Time for forecasts       6. Evidence reliance       7. Familiarity with and Anticipated Impact of Reproducibility Project       8. Estimate of perfect replication of effect sizes    2. Demographics:       1. age       2. gender       3. expertise: H index, title/rank (associate professor, full professor, etc.), number of publications, number of citations (all collected from both SCOPUS & Web of Science), nationality/region 3. Post-event Interview: Once results are available, all participants will be approached for posterior and recalled forecasts. We will collect the first set of posterior distributions when the first batch of results is published. Once all data are collected, we participants will be debriefed about study hypotheses and offered an opportunity to learn their forecast skill against the average of their peers. |
| INCENTIVIZING GOOD PERFORMANCE  Getting good forecasts requires providing strong motivations. We will create incentives in three different ways:   1. Top scores will be posted to <http://www.translationalethics.com/forecast-scoreboard/>. We will also attempt to partner with the journal that agrees to publish our manuscript for a feature article that reports on the project, and that lists the top 5 forecasters in each expert stratum by name - provided each expert provides written consent to be de-anonymized. 2. The top two expert forecasters in each stratum will be offered awards of $200 and $50 USD, respectively. The top two novice forecasters will be offered $50 and $25, respectively. 3. We will maintain a website that lists all participants in code, and that shows each individual’s Brier skill score, and the position of their score on a distribution of brier skill scores. Individuals will be told their code so that they can check their forecast performance against others. |
| PRIMARY ANALYSIS  We will calculate the Brier scores of each individual forecaster, and present a distribution. A Brier score is a proper scoring rule – meaning it incentivizes honest reporting of subjective beliefs – which measures the accuracy of a forecast: BS = 1/N $\sum_{i=1}^{N} \left( f_{i}-o_{i} \right)^{2}$ where *f_i_* is a forecast and *o_i_* is a realized outcome for each of i events.[12] We will decompose into calibration = 1/N $\sum_{i=1}^{N} {1/n_{i}\left( f_{i}-\bar{o}_{i} \right)}^{2}$, and discrimination = 1/N $\sum_{i=1}^{N} 1/n_{i}(\bar{o}_{i}\left( 1-\bar{o}_{i} \right))$.[13] We will construct calibration curves for individual forecasters. Our null hypothesis is that mean Brier score 95% confidence intervals exclude Brier score for a matched sample size of hypothetical forecasters predicting same trials using a random number generator. We will also measure the Brier Skill Score for individuals: BSS = 1 – BS/BS_ref_ where BS_ref_ is the base rate for the reference class or of a confidence interval. The sample variance can be used when is BS_ref_ unknown.[14]  Primary endpoint   1. Replicability rate measures: The brier scores for aggregate measures will be slightly higher than .25 (note: .25 is the equivalent of a 50-50 prediction meaning “I don’t know” or a random guess). The mean forecaster will perform slightly worse than base-rates. Forecasts will be skewed toward predicting more successful replications than observed, but less than statistically expected. The order of successful predictions of aggregate replicability rates will be: 2. Effect sizes at least as large (Brier of about .20 to .25) 3. Significance (p-values) (Brier of about .25)   An a priori power analysis suggests we need a sample size of 34 to detect if expert Brier scores are different than a constant (e.g. random guessing (BS = .25) or a base rate (e.g. 80% of significant effects in cancer phase 3 trials favor the new treatment[15]) by a moderate degree (difference from a constant of Cohen’s d = .5) assuming power = .80 and alpha = .05.   1. Individual study measures: The Brier scores for individual studies will be slightly lower than .25. Forecasts will predict more successful replications than observed. Brier scores will be lower (forecasts will be more successful) for forecasting the direction of the difference between effect sizes (Outcome2) compared to forecasting significant differences (Outcome1). Base-rates will outperform the typical forecaster, but aggregating the top-tier of forecasters will beat base-rates. |
| SECONDARY ANALYSIS ON FORECAST SKILL^[[4]](#footnote-4)^  An a priori power analysis suggests we need a sample size of 34 per group to detect if detect if the experts differ from (d = .5) the novice group assuming power = .80 and alpha = .05, and we need about 180 total respondents to detect a moderate difference (f = .25) between all four strata. As analysis is exploratory, we do not intend to correct for multiple hypothesis testing.   1. Expert strata analysis: 2. The order of predicted replication rates will be: 3. Novices/Non-experts (Should be optimistic since and less attuned to the “replicability crisis” across scientific disciplines) 4. Originators (if available- should be optimistic due to their investment in these results, but more realistic than novices) 5. Independent Experts (Should be less biased due to independence from findings) 6. Replicators (if available- theoretically well attuned to flawed research design and practices) 7. The order of forecast skill will be: 8. Independent Experts (should have sufficient expertise and less biased by own work) 9. Originators (if available- should have sufficient expertise, but more myside bias) 10. Replicators (if available- assumes lab skills are higher than knowledge for this group) 11. Novices/Non-experts (assuming expertise and forecast skill are related, they have the least expertise) 12. The order of variability in forecasts will be: 13. Novices/Non-experts (assuming expertise and forecast skill are related, there should be more variability for those with the least knowledge) 14. Independent Experts 15. Replicators (if available- assumes replicators are well attuned to good and bad study design and difficulty of carrying out certain studies) 16. Originators (if available- assuming expertise and forecast skill are related, there should be less variability in recognizing real empirical relationships and flawed study design by those who have devoted their lives to studying these topics) 17. Expertise will have an inverse U-shaped (∩) relationship with forecasting success. People with low expertise will perform poorly due to inadequate information, and people with high expertise will perform poorly due to maladaptive beliefs (e.g. confirmation bias). The correlation between perceived expertise and Brier score will be ranked as follows:      1. Originators (if available- should perform when they claim higher expertise; have motivation to honestly claim high expertise for some, but not all topics) 2. Independent Experts (may be less discriminating in expertise ratings than originators since they don’t have personal relationships with any of the studies) 3. Replicators (if available) 4. Novices/Non-experts (should have consistently low expertise ratings) 5. Predictors of Forecast Skill: We will test the relationship between each demographic and scale variables and Brier Skill Score, secondarily looking at components of calibration and discrimination. The individual differences should influence the forecasts in the following order (from most to least influential). 6. More extreme updaters: Those who update more extremely relative their will have more accurate forecasts^[[5]](#footnote-5)^. 7. Pace of progress: Those who believe medical technology should progress quickly (slowly) will have more optimistic (pessimistic) forecasts. 8. Evidence reliance: 9. Those who rate the methodology of the replication attempt as important will be pessimistic. 10. Those who cite specific trial outcomes as evidence will be worse forecasters, and those who cite treatment or disease type (base rates) will be better forecasters. 11. Expertise: Those will high (self-reported and actual) expertise will be more accurate; actual expertise (H- & M-indices) will be more predictive of forecasting skill. 12. Confidence will be weakly correlated with forecasting skill. We expect a hard-easy effect where forecasters are overconfident on hard items and underconfident on easy items. 13. Demographics     1. We will also test methods of identifying forecasting skill early (prior to target event realization) the relationship between individual forecaster’s Brier scores and their average squared distance to the median compared to other baselines (mean, expectation due to chance, expectation due to base rates if available) - we hypothesize that this will lead to a strong relationship with forecast skill. 14. Aggregation of Forecasts 1: We will test whether different aggregation approaches give better forecast performance (i.e. lower Brier scores). First, to determine the relative value of using combined forecasts to make decisions, we will construct a curve of showing the relationship between Brier scores and number of aggregated forecasts. Specifically, the curve will reflect n=0 aggregation (i.e. average Brier scores of individual forecasters); n=1 (i.e. average Brier score when two forecasters forecasts are randomly aggregated); n=4 (i.e. average Brier score when 5 randomly selected forecasts are aggregated); and n=9 (i.e. average Brier score when 10 randomly selected forecasts are aggregated). We will also use transformations to make predictions more extreme.[16,17] 15. Predictors and Aggregation: Using individual differences and demographic variables that show low skill identified in step 1, we will test the competitiveness of aggregated low skill forecasters with the individual scores of high skill forecasters. For instance, suppose we find a relationship between high M-index and high forecast skill. We will then test whether, if we combine forecasts from 2, 3, 5 and 10 low M-index forecasters, on average the forecast outperforms the average forecast of high M-index forecasters.   After we have completed forecast scoring, we will also rank all individual forecasters by their Brier skill scores, and randomly select individuals within tiers of upper, middle, and lower thirds. We will test whether combined forecasts of 2, 3, 5 and 10 “low skill” forecasters can, on average, beat the forecast of randomly selected individuals from each skill tier. Brier scores will be used; as well, they will be decomposed into discrimination and calibration. |
| SECONARY ANALYSIS ON UPDATING AND RECALL   1. Updating: To determine the extent to which researchers properly update belief (as opposed to stubbornly clinging to their priors - or abandoning them), we will perform all primary and secondary analyses, scoring elicited posteriors against proper posteriors (calculated on the basis of Bayes’ Theorem using a beta distribution). |
| ETHICS  Issues: This study presents two human subject issues:  1. Subject burden: this study will require a 30-minute intake interview for approximately 110 researchers and 50 students; this will be followed up a year later or so with a debrief interview lasting no more that 20 minutes. This is a large commitment of time for busy researchers.  2. Confidentiality: What a researcher believes about an experiment or his or her forecast skills constitute sensitive information. We will strictly protect the confidentiality of all participants as well as their scores, as explained by the measures below  Protections:  1. General: Protocol will be submitted for independent ethical review prior to initiation. Informed consent will be obtained from all participants prior to data collection, and documented upon signing the questionnaire or initialing a consent box in the electronic interface.  2. Subject burden: We will minimize burden by minimizing sample size and streamlining interviews. Informed consent will be obtained; as scientific researchers are not a vulnerable population, we are confident they can protect their interests by deciding whether to participate. No students who are approached for participation will be enrolled in a class taught by the individuals on this protocol.  3. Confidentiality: All participants will receive a numeric identifier that is used to label all responses. The key linking participant identity to data will be maintained in a locked filing cabinet and a password protected computer. The key will be maintained only to enable recontact of investigators in the event of a follow-up study. Key and all identifying information will be destroyed within five years of data collection. All identifying information will be maintained in strict confidence.  The ‘best’ forecaster will be identified by the research team and notified as such. He or she will be given the option of being de-identified and profiled on the STREAM website. His/her permission to de-identify will be documented with a signature. |
| APPENDICES   1. Map of survey versions 2. Sample information sheet for Replication Study 3. List of replication studies |

**Protocol Appendix**

**Survey Versions**

1. Expert survey 1
   1. Studies 15, 21, & 44
2. Expert survey 2
   1. Studies, 19, 29, & 39
3. Expert survey 1, incentive pilot:
   1. Studies 15, 21, & 44
   2. included option to list email for us to send C$20 Amazon gift card or donate $20 to Canadian Cancer Society
4. Expert survey 2, incentive pilot:
   1. Studies, 19, 29, & 39
   2. included option to list email for us to send C$20 Amazon gift card or donate $20 to Canadian Cancer Society
5. Novice survey 1
   1. Studies 15, 21, & 44
   2. Exclude: Editor question
   3. Include:
      1. Cancer class or lab semesters question
      2. Add email to obtain results

**Sample Information Sheet**

| **Coadministration of a tumor-penetrating peptide enhances the efficacy of cancer drugs**  Sugahara, K. N., Teesalu, T., Karmali, P. P., … Ruoslahti, E.[18]  **Link to original paper:** <http://www.ncbi.nlm.nih.gov/pmc/articles/PMC2881692/> **Link to replication plan:** <http://elifesciences.org/content/4/e06959>  In the above study, the authors showed that a tumor-penetrating peptide, iRGD, when given to mice with orthotopic human prostate tumors, resulted in significantly greater anti-tumor activity for the small molecule drug, doxorubicin, as compared with animals given doxorubicin without the tumor-penetrating peptide (see arrows). The following 3 questions refer to the replication of this effect.  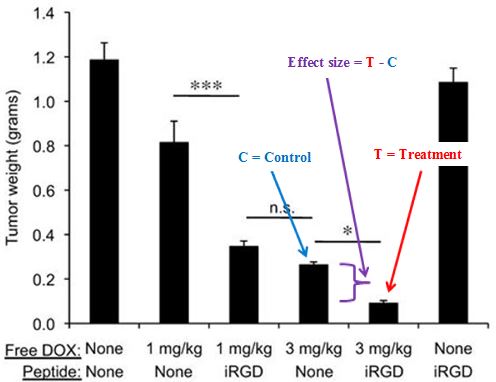 | | |
| --- | --- | --- |
|  | **Subjective Probability** (%) | **Confidence** (%) |
| 4. *Based on the claims of this paper*, how likely is it that the replication will also be **significant (at p ≤ .05)**? |  |  |
| 5. *Based on the claims of this paper*, how likely is it that the **effect size** of the replication will be **as large or larger than the original effect size**? |  |  |

**Original Studies**[18–23] **Undergoing Replication**

|  |  |  |  |  |
| --- | --- | --- | --- | --- |
| Study # | First Author | Title | Journal | Year |
| 15 | Sugahara | Coadministration of a tumor-penetrating peptide enhances the efficacy of cancer drugs | Science | 2010 |
| 21 | Sirota | Discovery and preclinical validation of drug indications using compendia of public gene expression data | Science | 2011 |
| 44 | Berger | Melanoma genome sequencing reveals frequent PREX2 mutations | Nature | 2012 |
| 19 | Delmore | BET bromodomain inhibition as a therapeutic strategy to target c-Myc | Cell | 2011 |
| 29 | Dawson | Inhibition of BET recruitement to chromatin as an effective treatment for MLL-fusion leukaemia | Nature | 2011 |
| 39 | Willingham | The CD47-signal regulatory preotein alpha (SIRPa) interaction is a therapeutic target for human solid tumors | PNAS | 2012 |

**References**

1. Collaboration OS. Estimating the reproducibility of psychological science. Science. 2015;349: aac4716. doi:10.1126/science.aac4716

2. Begley CG, Ellis LM. Drug development: Raise standards for preclinical cancer research. Nature. 2012;483: 531–533. doi:10.1038/483531a

3. Prinz F, Schlange T, Asadullah K. Believe it or not: how much can we rely on published data on potential drug targets? Nat Rev Drug Discov. 2011;10: 712–712. doi:10.1038/nrd3439-c1

4. Steward O, Popovich PG, Dietrich WD, Kleitman N. Replication and reproducibility in spinal cord injury research. Exp Neurol. 2012;233: 597–605. doi:10.1016/j.expneurol.2011.06.017

5. Tsilidis KK, Panagiotou OA, Sena ES, Aretouli E, Evangelou E, Howells DW, et al. Evaluation of Excess Significance Bias in Animal Studies of Neurological Diseases. PLOS Biol. 2013;11: e1001609. doi:10.1371/journal.pbio.1001609

6. Sena ES, Worp HB van der, Bath PMW, Howells DW, Macleod MR. Publication Bias in Reports of Animal Stroke Studies Leads to Major Overstatement of Efficacy. PLOS Biol. 2010;8: e1000344. doi:10.1371/journal.pbio.1000344

7. Lewandowsky S, Oreskes N, Risbey JS, Newell BR, Smithson M. Seepage: Climate change denial and its effect on the scientific community. Glob Environ Change. 2015;33: 1–13. doi:10.1016/j.gloenvcha.2015.02.013

8. Errington TM, Iorns E, Gunn W, Tan FE, Lomax J, Nosek BA. An open investigation of the reproducibility of cancer biology research. eLife. 2014;3: e04333. doi:10.7554/eLife.04333

9. Speirs-Bridge A, Fidler F, McBride M, Flander L, Cumming G, Burgman M. Reducing Overconfidence in the Interval Judgments of Experts. Risk Anal. 2010;30: 512–523. doi:10.1111/j.1539-6924.2009.01337.x

10. Haran U, Ritov I, Mellers BA. The role of actively open-minded thinking in information acquisition, accuracy, and calibration. Judgm Decis Mak. 2013;8: 188–201.

11. Tetlock PE. Expert Political Judgment. In: Princeton University Press. Available: http://press.princeton.edu/titles/7959.html

12. Brier GW. Verification of forecasts expressed in terms of probability. Mon Weather Rev. 1950;78: 1–3. doi:10.1175/1520-0493(1950)078<0001:VOFEIT>2.0.CO;2

13. Murphy AH. A New Vector Partition of the Probability Score. J Appl Meteorol. 1973;12: 595–600. doi:10.1175/1520-0450(1973)012<0595:ANVPOT>2.0.CO;2

14. Weigel AP, Liniger MA, Appenzeller C. The Discrete Brier and Ranked Probability Skill Scores. Mon Weather Rev. 2007;135: 118–124. doi:10.1175/MWR3280.1

15. Djulbegovic B, Kumar A, Soares HP, Hozo I, Bepler G, Clarke M, et al. Treatment Success in Cancer: New Cancer Treatment Successes Identified in Phase 3 Randomized Controlled Trials Conducted by the National Cancer Institute–Sponsored Cooperative Oncology Groups, 1955 to 2006. Arch Intern Med. 2008;168: 632–642. doi:10.1001/archinte.168.6.632

16. Baron J, Mellers BA, Tetlock PE, Stone E, Ungar LH. Two Reasons to Make Aggregated Probability Forecasts More Extreme. Decis Anal. 2014;11: 133–145. doi:10.1287/deca.2014.0293

17. Karmarkar US. Subjectively weighted utility: A descriptive extension of the expected utility model. Organ Behav Hum Perform. 1978;21: 61–72. doi:10.1016/0030-5073(78)90039-9

18. Sugahara KN, Teesalu T, Karmali PP, Kotamraju VR, Agemy L, Greenwald DR, et al. Coadministration of a tumor-penetrating peptide enhances the efficacy of cancer drugs. Science. 2010;328: 1031–1035. doi:10.1126/science.1183057

19. Sirota M, Dudley JT, Kim J, Chiang AP, Morgan AA, Sweet-Cordero A, et al. Discovery and preclinical validation of drug indications using compendia of public gene expression data. Sci Transl Med. 2011;3: 96ra77. doi:10.1126/scitranslmed.3001318

20. Berger MF, Hodis E, Heffernan TP, Deribe YL, Lawrence MS, Protopopov A, et al. Melanoma genome sequencing reveals frequent PREX2 mutations. Nature. 2012;485: 502–506. doi:10.1038/nature11071

21. Delmore JE, Issa GC, Lemieux ME, Rahl PB, Shi J, Jacobs HM, et al. BET Bromodomain Inhibition as a Therapeutic Strategy to Target c-Myc. Cell. 2011;146: 904–917. doi:10.1016/j.cell.2011.08.017

22. Dawson MA, Prinjha RK, Dittmann A, Giotopoulos G, Bantscheff M, Chan W-I, et al. Inhibition of BET recruitment to chromatin as an effective treatment for MLL-fusion leukaemia. Nature. 2011;478: 529–533. doi:10.1038/nature10509

23. Willingham SB, Volkmer J-P, Gentles AJ, Sahoo D, Dalerba P, Mitra SS, et al. The CD47-signal regulatory protein alpha (SIRPa) interaction is a therapeutic target for human solid tumors. Proc Natl Acad Sci. 2012;109: 6662–6667. doi:10.1073/pnas.1121623109

1. This number has since been reduced to 29 due to budgetary constraints. [↑](#footnote-ref-1)
2. The following protocol is the version that was implemented after piloting of an earlier, IRB-approved protocol. [↑](#footnote-ref-2)
3. We did not pursue these additional strata because we anticipated great difficulty accessing a sufficient sample of originators, after being advised about the controversial nature of the project and the fact that many felt over-extended due to time spent gathering and recreating original materials. Ultimately, without the originator stratum and due to their access to results, we decided not to pursue the replicators stratum either. [↑](#footnote-ref-3)
4. Analyses might not all be included in a single manuscript. We are not able to conduct some of the analyses described due to the lack of variability in observed replication results and since we excluded a couple of secondary expert strata. [↑](#footnote-ref-4)
5. We cannot measure belief updating until all of the replication results are announced and we collect posterior forecasts. [↑](#footnote-ref-5)
